# Congenital and inherited neurologic diseases in dogs and cats: Legislation and its effect on purchase in Italy

**DOI:** 10.14202/vetworld.2016.437-443

**Published:** 2016-05-06

**Authors:** Annamaria Passantino, Marisa Masucci

**Affiliations:** Department of Veterinary Sciences, University of Messina, Polo Universitario Annunziata, 98168 Messina, Italy

**Keywords:** buy/sell, cat, dog, hereditary disease, nervous system

## Abstract

Many of the congenital neurologic diseases can result in incapacity or death of the animal. Some of them, such as idiopathic epilepsy and hydrocephalus, exhibit breed or familial predisposition and a genetic basis was proved or suggested. Some diseases can be presumptively diagnosed after a detailed signalment (breed predisposition), history (e.g. family history because many of these defects have familial tendencies), and through physical exam; other diagnostic methods (radiography, computed tomography, magnetic resonance, electrophysiologic tests, etc.) can provide supportive evidence for the congenital defect and help to confirm the diagnosis. Some cases can lead to civil law-suits when the lesions are congenital, but not easily recognizable, or when the lesions are hereditary but tend to became manifest only after some time (more than 12 months after the date of purchase, e.g., after the vice-free guarantee period has expired). Moreover, quite frequently an early diagnosis is not made because there are delays in consulting the veterinarian or the general practitioner veterinarian does not perceive subtle signs. This study was designed to focus on the medico-legal aspects concerning the buying and selling in Italy of dogs and cats affected by congenital and hereditary neurologic diseases that could constitute vice in these animals. While adequate provisions to regulate in detail the various aspects of pet sale have still to be drawn up by legislators, it may be helpful to involve breeders, by obliging them by contract to extend guarantees in the case of hereditary lesions, including neurologic diseases.

## Introduction

To most European citizens, companion animals become more than just animals in the home. Cats, dogs, and other companion animals often occupy the status of the beloved family member. Despite this status which most of these animals gain in the home, they are legally considered mere goods [1] or commodities during the sales process (purchase). Indeed, companion animals have no independent status or personhood in the legal world. In the eyes of the law, animals are property: They are goods to be bought and sold, acquired, and maintained.

The term “goods” is meant to afford buyers and sellers certain rights and responsibilities in the transaction. The terms “possession” and “property” are often used synonymously. Nevertheless, there is an important difference. The term “property” (*dominium, proprietas*) refers to the right to dispose of a thing, while the term “possession” (*possessio*) designates the actual power over a thing.

The Italian Civil Code (article 812) at present considers animals as *res*, i.e., a thing (as property) as opposed to a person, who has rights [2,3].

The purchase (*emptio venditio*) is a contract that obliges one party (*venditor*) to provide a thing (a commodity) and the other party (*emptor)* to provide payment. It is a bilateral contract since both parties are creditors and debtors at the same time - the *venditor* owes the commodity and the *emptor* owes the money.

The seller is always liable for defects of the commodities (including animals). For redhibitory defects (vice), however, he/she is only liable if he kept them secret on purpose or if he guaranteed a faultless product.

In veterinary legal medicine, the illness must be considered a vice [4] in those cases where it renders an animal unsuitable for its specified use or significantly reduces its value.

An animal, which is unsuitable for its specified use, must be affected with a behavioral character defect or illness. The illness causes a disturbance in normal organic functioning, which may be localized or generalized, following an anatomic alteration which will inevitably cause an appreciable permanent or temporary impairment.

To qualify as vice, however, illness must be [4,5]:

1. Pre-existing or have a pre-existing cause. It is clear that congenital and hereditary neurologic diseases can come under this heading since most of them are typically primary; for this reason, it is difficult to demonstrate that environmental factors have contributed to their phenotypization;
2. Hidden. This means that it cannot be discovered by an ordinary inspection or examination; or rather, it is not easily recognized at the moment of purchase, and it cannot be detected using the normal due diligence.
3. Serious or chronic so as to affect the use of the animal or such that, if the buyer knew of the disease, he/she would not enter into the contract. In fact, defect renders the thing sold unfit for the use for which it is intended or diminishes its fitness for the intended use to such an extent that the vendee would not have bought or would have given a lower price if he had been aware of the defect.

In dogs and cats, there are many neurologic conditions of proven or suspected hereditary origin, both congenital and non-congenital, which may constitute vice in the buying and selling of these animals.

These cases would be lead to civil law-suits when the lesions are congenital, but not easily recognizable, or when the lesions are hereditary but tend to became manifest only after some time (more than 12 months after the date of purchase, i.e., after the vice-free guarantee period has expired). Moreover, an early diagnosis quite frequently is not made because there are delays in consulting a veterinarian or the general practitioner veterinarian does not perceive subtle signs.

This study will present an overview of general contract law related to sales of dogs and cats as well as the rights and remedies of buyers under Italian law. Finally, the study will highlight some concerns facing buyers, especially when purchasing companion animals (dogs and cats) affected by congenital and hereditary neurologic diseases.

### Current Italian Law

In Italy, the purchase of animals is regulated by the Civil Code (article 1470 and subsequent) and dated back to 1942. These articles regulate the purchase of real property, and they are also adopted in the field of the purchase of animals.

In fact, the only article concerning animal purchase in Italy is article 1496 of the Italian Civil Code. It states that the guarantee against vices in the sale of animals is regulated by special laws or by local usage or, if these are lacking, by article 1490 of the Civil Code and subsequent. The latter reads as follows: “The seller is obliged to guarantee that the object sold is immune from vices which make it unsuitable for the usage to which it is destined or that decrease the value of it in an appreciable way.”

In Italy, only when the animal is affected by a serious or chronic vice that is pre-existing and not easily recognizable, will the buyer be able, with respect to the terms of expiry and to rules contained in article 1495 of the Italian Civil Code, to exercise one of the two legal actions foreseen by the Code, namely, redhibitory action or estimatory action (remedies of the vendee). “Redhibitory action” (*actio redhibitoria*) means rescission of sale, and “estimatory action” (*actio estimatoria* or *quanti minoris*) means reduction of the price (article 1492 Civil Code).

The time limits for rescission or exaction of proportionate reduction of the purchase price against the vendor under article 1495 are 8 days from the day of delivery and 1 year from the date of purchase.

### Congenital and Inherited Neurological Diseases

Physiologic nervous system development may be affected by inherited or congenital factors, e.g., *in* *utero* exposure to viral (parvovirus-induced cerebellar hypoplasia) or teratogenic substances (cranium bifidum, spina bifida, abnormal atlanto-occipital articulation, exencephaly, and hydrocephalus associated with griseofulvin therapy) [6,7].

Many kinds of anomaly result from pathologic nervous system development:

- Macroscopic malformations (hydrocephalus, cerebellar hypoplasia, lissencephaly, hydromyelia, syringomyelia, spinal dysraphism) [8-15];
- Microscopic lesions (involving the inner ear in congenital deafness and vestibular syndrome and the gray or white matter in degenerative diseases) [9,16,17];
- Alterations involving molecular structures only (decreased number of acetylcholine receptors in congenital myasthenia gravis, enzymatic deficiency in storage diseases, abnormalities of neurotransmitters or their receptors in narcolepsy) [9,18-24].

In some cases, the involvement of the nervous system results from malformations of the skull or the spine (meningocele and myelomeningocele in association with spina bifida, spinal cord compressions caused by hemivertebrae, butterfly vertebrae, transitional vertebrae, and malformation of the dens of the axis) [9,10,12,25-30].

In many cases, the clinical signs are apparent at birth and there is no progression of the disease, but sometimes, e.g., in degenerative diseases, the onset of symptoms occurs in the first few months or years of life, and they are slowly progressive and lead to the animal’s death (Table-1). Clinical signs related to idiopathic epilepsy and spinal cord compression due to congenital vertebral anomalies can develop in adult animals (Table-1) [25,31]. Mild neurologic signs, although present at birth, can be mistaken for the normal awkwardness of the puppies by their owner.

**Table-1:** Breeds affected by inherited neurologic diseases [9,10,34-38].

Disease	Dog	Cat
**Abiotrophies** Incidence: Rare Prognosis: Guarded to poor (there are different degrees of the disease, which is slowly progressive) Age of onset of clinical signs: 6 weeks to 5 years Clinical signs: Cerebellar ataxia, paresis/paralysis Diagnosis ante mortem: Genetic (American Staffordshire Terrier); in other breeds only post mortem	American Staffordshire Terrier, Australian Kelpie, Beagle, Bernese Mountain Dog, Blue Terrier, Bobtail, Border Collie, Breton Spaniel, Cairn Terrier, Chow Chow, Cocker Spaniel, Doberman Pinscher, Fox Terrier, German Shepherd Dog, Golden Retriever, Gordon Setter, Kerry Samoyed, Labrador Retriever, Lapland, Pointer, Poodle, Rhodesian Ridgeback, Saluki, Wire-haired Great Dane	Domestic, Siamese
**Ceroid lipofuscinosis** Incidence: Rare Prognosis: Guarded to poor Age of onset of clinical signs: 4 months to 9 years (most animal<2 years) Clinical signs: Personality change, visual impairment, ataxia, seizures Diagnosis ante mortem: Yes (genetic, histopathology of skin biopsies)	Australian Cattle Dog, Blue Heelers, Blue Heelers, Border Collie, Chihuahua, Cocker Spaniels, Dachshund, English Setter, Salukis, Tibetan Terrier, Yugoslavian Sheep Dog	Domestic, Siamese
**Congenital myasthenia gravis** Incidence: Rare Prognosis: Guarded Age of onset of clinical signs: 6-8 weeks Clinical signs: Weakness worsening after exercise Diagnosis ante mortem: Yes (electrodiagnostic tests, pharmacological tests, muscle biopsy)	Fox Terrier, Jack Russel Terrier, Samoyed, Springer Spaniel	Siamese
**Demyelinating diseases of peripheral nerves** Incidence: Rare Prognosis: Poor Age of onset of clinical signs: 3 weeks to 3 months Clinical signs: LMN paresis/paralysis Diagnosis ante mortem: Yes (peripheral nerve biopsy)	Alaskan Malamute, Golden Retriever, Tibetan Mastiff	Siamese
**Fucosidosis** Incidence: Rare Prognosis: Poor Age of onset of clinical signs: 6 months to 2 years Clinical signs: Various, predominantly behavioral and motor Diagnosis ante mortem: Yes (enzyme analysis, genetic tests)	Springer Spaniel	
**Gangliosidosis** Incidence: Rare Prognosis: Poor Age of onset of clinical signs: 2 months to 1.5 years Clinical signs: Vision deficits, lethargy, gait disturbances Diagnosis ante mortem: Yes (blood cytology, genetic tests)	Alaskan Husky (controlla denominazione), Beagle, German Shorthaired Pointer, Japanese Spaniel, Portuguese Water Dog, Shiba, Springer Spaniel	Domestic, Korat, Siamese
**Globoid cell leukodystrophy** Incidence: Rare Prognosis: Poor Age of onset of clinical signs: 5 weeks to 2 years Clinical signs: Cerebellar ataxia, paraparesis/plegia amaurosis, behavior disturbance Diagnosis ante mortem: Yes (genetic tests)	Basset Hound, Beagle, Blue Tick Coonhound, Cairn Terrier, Pomeranian, Poodle, West Highland White Terrier	Domestic
**Glucocerebrosidosis** Incidence: Rare Prognosis: Poor Age of onset of clinical signs: 6-8 months Clinical signs: Ataxia Diagnosis ante mortem: Yes (enzyme assay)	Silky Terrier	Abyssinian
**Glycogenosis** Incidence: Rare Prognosis: Poor Age of onset of clinical signs: 5 months to 1.5 years Clinical signs: Two forms: Early neonatal death or progressive neuromuscular weakness Diagnosis ante mortem: yes (genetic test in IV type in the cat)	German Shepherd Dog, Lapland Dog, Springer Spaniel	Domestic, Norwegian Forest Cat
**Hemivertebrae, Butterfly vertebrae** Incidence: Common Prognosis: Good to poor Age of onset of clinical signs: Variable Clinical signs: Asymptomatic or ataxia, paresis/paralysis due to acute or chronic spinal cord compression Diagnosis ante mortem: Yes (RX, CT)	“Screw-tailed” brachycephalic breeds: Boston Terrier, French and English Bulldog, Pug	
**Hydrocephalus** Incidence: Common Prognosis: Guarded Age of onset of clinical signs: Shortly after birth Clinical signs: Enlargement of the skull, open sutures, and fontanelles, behavioral changes, depression, seizures, amaurosis, ataxia Diagnosis ante mortem: yes (ultrasonography, TC, MR, EEG)	Toy breeds: Chihuahua, Maltese, Pomeranian, Yorkshire Terrier, Toy Poodle Brachycephalic breeds: Boston terrier, English Bulldog, Lhasa Apso, Pekingese, Pug	Siamese
**Hypomyelinating diseases** Incidence: Rare Prognosis: Guarded (not progressive and improvement can occur) Age of onset of clinical signs: Shortly after birth Clinical signs: Tremors Diagnosis ante mortem: No (post mortem histopathology)	Bernese Mountain Dog, Chow Chow, Dalmatian, Samoyed, Springer Spaniel, Weimaraner	
**Idiopathic epilepsy** Incidence: Very common in dogs, rare in cats Prognosis: Guarded Age of onset of clinical signs: 6 months to 5 years Clinical signs: Seizures Diagnosis ante mortem: Yes (by excluding other causes of seizures. No positive diagnostic signs can substantiate the diagnosis)	Beagle, Bernese Mountain Dog, Boxer, Cocker Spaniel, Collie, Dachshund, German Shepherd Dog, Golden Retriever, Irish Setter, Keeshond, Labrador Retriever, Poodle, Saint Bernard, Siberian Husky, Tervuren, Wire Fox Terrier	
**Inherited deafness** Incidence: Common Prognosis: Good *quoad vitam* Age of onset of clinical signs: Shortly after birth Clinical signs: Deafness Diagnosis ante mortem: Yes (electrodiagnostic tests)	Argentine Dogo, Australian Shepherd, Bobtail, Dalmatian, Doberman Pinscher, English Bulldog, English Setter, Foxhound, Great Dane, Great Pyrenees, Maltese, Pointer	*White blue- eyed cats*
**Leukodystrophies** Incidence: Rare Prognosis: Poor Age of onset of clinical signs: Few weeks to few years Clinical signs: Ataxia, paresis/paralysis, seizures Diagnosis ante mortem: No (post mortem histopathology)	Afghan Hound, Dalmatian, Kookier, Labrador Retriever, Poodle, Rottweiler, Scottish Terrier	
**Lissencephaly** Incidence: Rare Prognosis: Guarded Age of onset of clinical signs: Shortly after birth-12 months Clinical signs: Behavioral changes, seizures, amaurosis, visual deficit Diagnosis ante mortem: Yes (CT, MR)	Fox Terrier, Irish Setter, Lhasa Apso	Domestic
**Malformations of the dens of the axis** Incidence: Common Prognosis: Guarded Age of onset of clinical signs: <1 year Clinical signs: Cervical pain, tetraparesis/paralysis Diagnosis ante mortem: Yes (RX, CT)	Toy breeds	Domestic
**Mannosidosis** Incidence: Rare Prognosis: Poor Age of onset of clinical signs: 2-7 months Clinical signs: Facial dysmorphism, ataxia, tremors, altered behavior, seizures Diagnosis ante mortem: Yes (genetic tests)		Domestic, Persian
**Mucopolysaccharidosis** Incidence: Rare Prognosis: Poor Age of onset of clinical signs: 3-10 months Clinical signs: Progressive paresis Diagnosis ante mortem: Yes (urinary biochemical tests, genetic tests (in mucopolysaccharidosis VI of the cat)	Dachshund, Labrador Retriever, Doberman Pinscher, Plott Hound	Domestic, Siamese
**Narcolepsy** Incidence: Rare Prognosis: Good quoad vitam (the severe forms can be invalidating) Age of onset of clinical signs: <6 months Clinical signs: Recurring sudden attacks of sleep and/or loss of muscle tone Diagnosis ante mortem: Yes (clinical diagnosis, electrodiagnostic tests, genetic tests)	Doberman Pinscher, Labrador Retriever	
**Peripheral axonopathies** Incidence: Rare Prognosis: Guarded to poor Clinical signs: Tetraparesis/paralysis Diagnosis ante mortem: Yes (peripheral nerve histopathology)	Boxer, German Shepherd Dog, Rottweiler	
**Sfingomyelinosis** Incidence: Rare Prognosis: Poor Age of onset of clinical signs: 2-4 months Clinical signs: Ataxia, tremors, paresis/paralysis Diagnosis ante mortem: Yes (enzyme assay)	Boxer, Poodle	Balinese, Domestic Siamese
**Spina bifida/meningomyelocele** Incidence: No common Prognosis: Good (spina bifida only) guarded to poor (spina bifida with meningomyelocele) Age of onset of clinical signs: Shortly after birth Clinical signs: Spina bifida can be asymptomatic, meningomyelocele: incontinence, paraparesis/paralysis Diagnosis ante mortem: Yes (clinical signs, RX, CT)	English Bulldog	Manx Cat
**Spongiform degeneration of gray or white matter** Incidence: Rare Prognosis: Poor Age of onset of clinical signs: 1-6 months Clinical signs: Tremors, ataxia, behavioral changes, mental status alterations, visual deficit Diagnosis ante mortem: No (histopathology post mortem)	Bull Mastiff, Cocker Spaniel, Labrador Retriever, Rottweiler, Saluki, Samoyed, Silky Terrier	Birman, Egyptian Mau

LMN=Lower motor neuron, CT=Computed tomography, EEG=Electroencephalogram, MR=Magnetic resonance

Some congenital diseases may be diagnosed by physical examination because there are typical signs (e.g., hydrocephalus, myelomeningocele, and meningoencephalocele). Appropriate diagnostic tests are required for many congenital neurologic diseases (Table-1). In some cases, the diagnosis can be made only by excluding other causes (e.g., idiopathic epilepsy) or only after the death of the animal because it requires anatomic and histopathologic evaluations (e.g. degenerative diseases) (Table-1) [9,10].

Inherited neurologic diseases usually affect specific breeds (Table-1).

### An Approach to the Problem: Proposals

The authors propose to make eradication plans for hereditary and/or congenital neurologic disease official and, indeed, obligatory for legal and ethical reasons. Genetic selection giving preference to certain characteristics can result in hereditary neurologic defects, which may cause problems of varying entity and this shows a lack of respect animals as sentient beings [32]. In this context, there are emotional and psychological implications for the owner who is made aware of the fact that his animal suffers from hereditary and genetic neurologic defects.

To reduce the incidence of congenital and/or hereditary neurologic disease in pets, it would also be useful that:

- Practicing veterinarians discourage reproduction in animals with neurologic alterations for which inherited etiology is recognized or suspected.
- Breeders and geneticists work together on eradication of hereditary anomalies. Genetic tests, in particular, make possible a rapid, accurate, and early confirmation of diagnosis in sick animals even before the clinical signs are evident; the carriers can thus be removed from breeding programs [33]. When genetic tests are not available, detailed information on pedigrees will make identification of carriers possible so as to carry out selective and rational breeding.

Another useful development could be to enhance breeders’ responsibility by enforcing the inclusion of a lengthening of guarantee time in the case of hereditary defects, including neurologic defects, which may only become evident after the age of 12 months (Table-1), that is, beyond the validity of the guarantee.

## Conclusions

Cooperation among dog breeders, researchers, prospective purchasers, and purebreed dog organizations at all levels is essential if genetically healthy dogs are to become a reality.

Breeders should understand the implications of genetic diseases recognized as affecting their breeds and take steps to breed only those dogs/cats that will minimize the propagation of unwanted characteristics.

Prospective buyers should be made aware of the genetic diseases related to the breed they are considering. They should also ask a physical exam and test results or genetic histories for the animals they are planning to purchase.

Veterinarians should inform owners, breeders, and prospective breeders about congenital/hereditary neurologic diseases.

## Authors’ Contributions

AP and MM generated the concept, collected materials, draft, and revised the manuscript. Both authors read and approved the final manuscript.
